# Photoprotection by Workwear: Ultraviolet Protection Factors for Artificial Radiation from Welding Arcs

**DOI:** 10.1111/php.13194

**Published:** 2020-01-22

**Authors:** Stefan Bauer

**Affiliations:** ^1^ Federal Institute for Occupational Safety and Health (BAuA) Dortmund Germany

## Abstract

Photoprotection by clothing is one of the most effective protective measures against solar ultraviolet radiation (UVR), and the related concept of UV protection factors (UPF) for fabrics has proven itself for decades. Although immense work has been carried out regarding factors influencing textile sun protection, the idea of UPFs for artificial UVR, if necessary with different action spectra, is payed little attention. In this work, a modified UPF equation is proposed utilizing welding arcs and the ICNIRP action spectrum. These welding UPFs (wUPF) were calculated for some workwear as a function of welding power, technique and welded material. No wUPF welding power dependence existed. By means of normalized standard emission spectra, that were further clustered with regard to welding technique, it was found that the wUPF depends exclusively on the fabric’s transmittance and the ICNIRP weighting function yielding a reduced wUPF formula. Finally, a comparison of solar and welding UPFs, based on either their full or reduced equations, is discussed.

## Graphical abstract

Ultraviolet protection factors (UPF) for clothing are based on the solar ultraviolet radiation (UVR) spectrum in conjunction with the CIE erythema weighting function and consider the textile’s UVR transmittance for wavelengths above 290 nm. However, workwear exposed to welding arcs that emit significant UVR at shorter wavelengths has to fulfill additional textile photoprotection criteria. Therefore, a welding UPF (wUPF) is proposed taking into account the welding arc’s full UVR emission spectrum and ICNIRP’s relative spectral effectiveness for UVR hazards. Analyzing the new protection factor regarding welding power, technique and welded material led to a reduced wUPF equation.

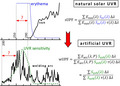

## Introduction

In 2012, natural as well as artificial UVR was classified by the International Agency for Research on Cancer (IARC) as a group I carcinogen to humans 1. One of the major protective measures to reduce risks for detrimental health effects from UVR sources is wearing clothes. With regard to sunscreens, there are several advantages of textile photoprotection, for example, clothing is easier to apply or provides a more reliable combined UVA and UVB protection, if rated with a high UPF 2. In addition, several factors can influence photoprotection by clothing like the type of fabric material, grammage, color or laundering 3, 4, 5. Introduced by Gies et al. 6 in 1994, the level of a fabric’s solar UVR protection can be determined by calculating its ultraviolet protection factor (UPF) according to

(1) $$\text{sUPF}=\frac{\sum_{290\;\text{nm}}^{400\;\text{nm}}\;E_{\text{sun}}\left(\lambda \right)\;S_{\text{er}}\left(\lambda \right)\Delta \lambda }{\sum_{290\;\text{nm}}^{400\;\text{nm}}\;E_{\text{sun}}\left(\lambda \right)\;S_{\text{er}}\left(\lambda \right)\;\tau \left(\lambda \right)\Delta \lambda }$$

Based on the solar spectral irradiance, *E*_sun_(λ), weighted by the International Commission on Illumination (CIE) erythema reference action spectrum, *S*_er_(λ) 7, this hereafter called solar UPF (sUPF) reflects the ratio of incident to transmitted (*τ*: transmittance) natural UVR through the fabric.

The success of the sUPF concept not only is discernible by its widespread use all over the world, but also by its implementation in several national or international standards, for example, in the Australian/New Zealand (AS/NZS 4399), the European (EN 13758) or the US American standard (AATCC Test Method 183). A comparison of these standards can be found in an overview article by Gies 8. In the following, the spectral irradiance of the Albuquerque summer sun will be used from EN 13758‐1 9 yielding a constant weighted solar irradiance of 0.22 Wm^−2^ in the numerator of Eq. (1).

### Artificial UVR

Research on photoprotection by clothing has been carried out for decades, but scientific work applying the UPF concept to artificial UVR sources is rare. Stempień et al. 10 measured UV protection coefficients, *η* = log *τ* ^−1^, using a medium pressure mercury arc lamp, but avoided the sUPF equation because of its limited wavelengths ranging from 400 to 290 nm, only. There is an approach by Aguilera et al. 11, who determined sUPFs for four different action spectra—previtamin D_3_, nonmelanoma skin cancer, photoimmunosuppression and photoaging—but the sun was still used as the UVR source.

In contrast to artificial UVR sources like xenon arc or germicidal lamps, UVR emission of certain welding arcs can be significantly high for wavelengths λ < 250 nm, and actinic irradiances, *E*_eff_, can range up to 200 Wm^−2^ (measurement distance: 50 cm). Recently, the welding arc’s UVR emission has been investigated thoroughly with regard to welding power, technique and welded material 12; therefore, the welding arc was chosen as the source of artificial UVR for this work. This allows testing photoprotection by workwear as a function of a wide range of actinic irradiances and varying spectral distributions due to different combinations of welding technique and welded material. In addition, welders are usually well protected by their special clothing, but people nearby or passing the welding workplace typically are dressed with “general” workwear that may not be made of, for example, heavyweight cotton or leather.

### Action spectra

**Figure 1:**
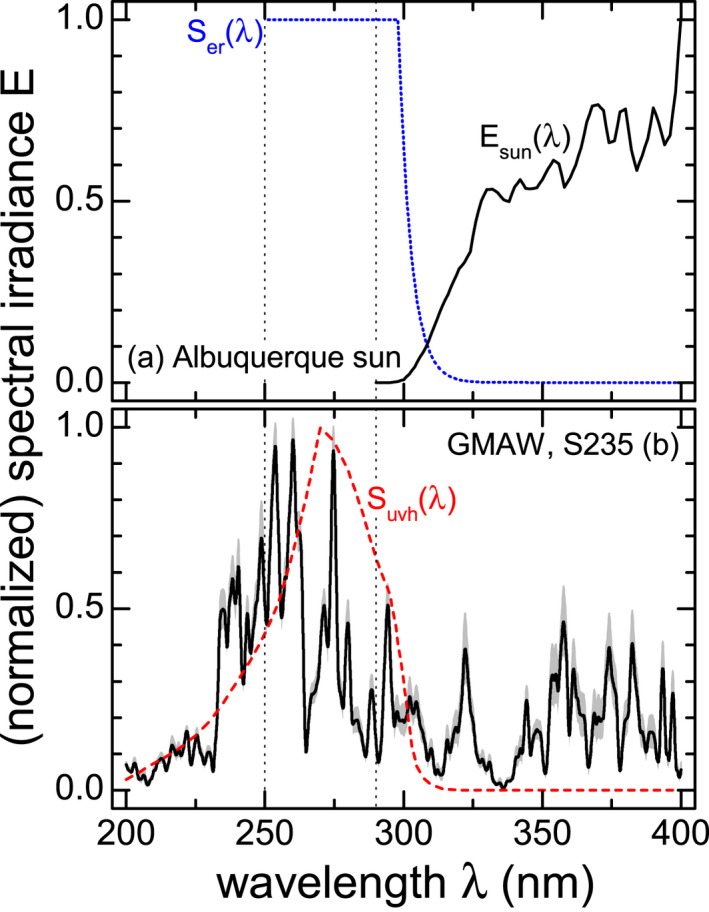
(a) Ultraviolet spectral irradiance of the Albuquerque summer sun, *E*_sun_(λ), according to EN 13758‐1 (not normalized, solid line) 9, and CIE erythema reference action spectrum, *S*_er_(λ), (short dotted line) 7. (b) Normalized standard welding emission spectrum, *ε*_(_ *_m_*_,_ *_n_*_)_(λ), for gas‐shielded metal arc welding (GMAW) of structural steel (S235, solid line). The standard deviation ± *s* for the arithmetic mean of 36 (p‐) MAG and (p‐) CMT welding spectra is shown as a shaded area. The ICNIRP relative spectral effectiveness of UVR, *S*_uvh_(λ), is depicted as a dashed line 13. In addition, vertical dotted lines represent constant wavelengths of 250 nm and 290 nm.

Comparing *E*_sun_(λ) with the standard UVR emission spectrum for gas‐shielded metal arc welding (GMAW) of structural steel (S235) in Fig. 1 (a) and (b), completely different spectral distributions are present with a large amount of the welding arc’s spectral irradiance emitted below λ < 290 nm, the cutoff wavelength of the solar UVR spectrum.

Consequently, the welding arc’s UVR emission in the wavelength range from 290 to 250 nm would not be taken into account in the sUPF calculation according to Eq. (1). Furthermore, UVR with even shorter wavelengths, λ < 250 nm, must also be considered in a modified version of the sUPF because significantly high emissions lines, up to 0.5 of normalized irradiance, can appear in arc spectra of certain welding processes 12, Fig. 1. CIE’s relative spectral effectiveness for erythema development, *S*_er_(λ), has not been derived with artificial light sources that have substantial UVC emissions 13. In addition, *S*_er_(λ) is constant (equal to 1) below 298 nm disregarding the wavelength dependent UVC absorption of human skin 14. However, epidermal UVC attenuation can be quite large, but it is unclear how wavelengths below 280 nm contribute, for example, by synergetic effects with other wavelength regions, to erythema or skin cancer. The relative spectral effectiveness for UVR hazards recommended by the International Commission on Non‐Ionizing Radiation Protection (ICNIRP), hereafter denoted as *S*_uvh_(λ) 13, see Fig. 1 (b), covers most of the UV spectral region and applies to 180 nm ≤ λ ≤ 400 nm. It must be noted that this weighting function is an envelope of detrimental UVR effects to both the human skin and eye.

**Figure 2:**
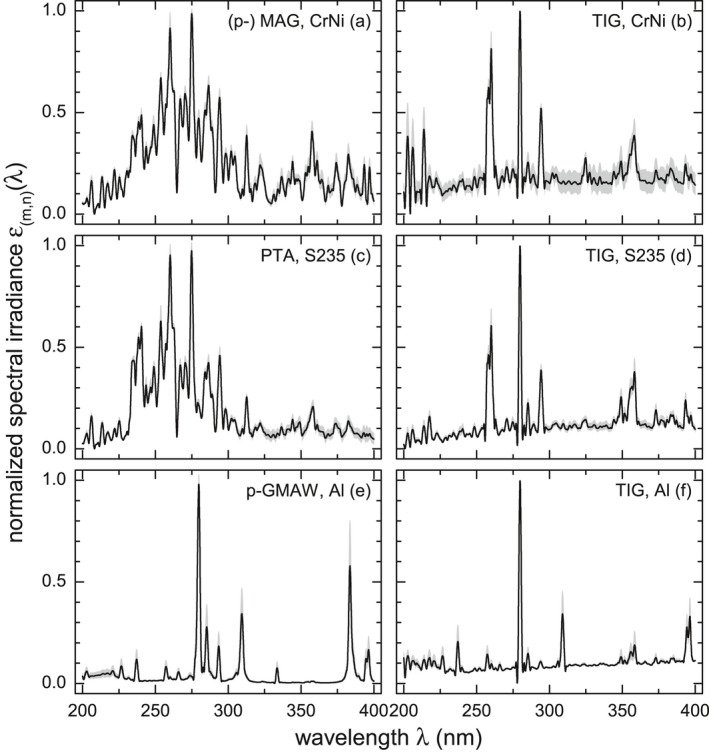
Normalized standard welding emission spectra, *ε*_(_ *_m_*_,_ *_n_*_)_(λ), of several pulsed and standard welding techniques for stainless (CrNi) and structural steel (S235) as well as for aluminum (Al). The standard deviations ± *s* due to averaging of (a) 25, (b) 10, (c) 8, (d) 11, (e) 30 and (f) 19 different spectral irradiances, depending on welding power *P*, are shown as shaded areas.

Another problem arises with *S*_er_(λ) applied at workplaces. The CIE erythema reference action spectrum is one of the most established weighting functions, but it is not directly related to exposure limit values (ELVs). Usually, the smallest value of the minimal erythemal dose (MED) for the most sensitive Fitzpatrick skin phototype I 15 is used to derive maximum permissible exposure durations. This approach reflects the worst case scenario because of the range of possible 1 MED values, even for the same skin phototype. CIE and ICNIRP recognize 200 Jm^−2^ as the smallest 1 MED value for skin phototype I 16, 17. Concerning artificial UVR, ICNIRP recommends limiting the daily effective radiant exposure at workplaces to *H*_eff_ = 30 Jm^−2^ 13, and this dose is used as legal ELV (or Threshold Limit Value, TLV), for example, in Europe or in America. In contrast to 1 MED, this effective or actinic radiant exposure, *H*_eff_, is based on the ICNIRP action spectrum for UVR hazards.

Overall, *S*_uvh_(λ) is a promising candidate for the determination of a modified UPF against artificial UVR from welding arcs. Wolska et al. 18 draw similar conclusions and introduced the actinic UPF (AUPF) that is the basis for the welding UPF (wUPF). Finally, it is worth mentioning that CIE recommends *S*_er_(λ) in the sUPF assessment just because “… it is slightly easier to use, and has been used in the past more widely in fabric testing applications” 19, and that “… there is such variation in the human response that the action spectrum can never be exact …” 20.

### Welding UPF

To adapt the sUPF equation to a welding workplace situation, first, the CIE reference erythema action spectrum, *S*_er_(λ), is replaced by the ICNIRP weighting function, *S*_uvh_(λ), accompanied by an extension of the lower summation limit to 200 nm. Second, the standard Albuquerque solar spectral irradiance is substituted by the welding arc’s UVR emission. Its spectral irradiances, *E*_(_ *_m_*_,_ *_n_*_)_(λ,*P*), depend on welding power *P*, welding technique *m* and welded material *n*. The associated *S*_uvh_(λ) weighted actinic irradiances, *E*_eff__(*m*__,__*n*)_(*P*), can be well described mathematically 12. In conjunction with the fabric’s transmittance, *τ*_*k*_(λ), the equation for the proposed wUPF can be written according to

(2) $${\text{wUPF}}_{k\left(m,n\right)}\left(P\right)=\frac{\sum_{200\;\text{nm}}^{400\;\text{nm}}\;E_{\left(m,n\right)}\left(\lambda ,P\right)\;S_{\text{uvh}}\left(\lambda \right)\;\Delta \lambda }{\sum_{200\;\text{nm}}^{400\;\text{nm}}\;E_{\left(m,n\right)}\left(\lambda ,P\right)\;S_{\text{uvh}}\left(\lambda \right)\;\tau_{k}\left(\lambda \right)\;\Delta \lambda }=\frac{E_{\text{eff}\left(m,n\right)}\left(P\right)}{E_{\text{eff,}\tau |k\left(m,n\right)}\left(P\right)}$$

The subscript index *k* refers to different fabrics. The denominator of wUPF_*k*(_ *_m_*_,_ *_n_*_)_(*P*) in Eq. (2) can be regarded as the biologically effective irradiance that is transmitted through the fabric, *E*_eff,*τ*|*k*(_ *_m,n_*_)_(*P*). At a first glance at Eq. (2), the wUPF adaption does not seem to be an advance in the field of photoprotection by clothing, but as it will be shown hereinafter, a reduced version of wUPF*_k_*_(_ *_m_*_,_ *_n_*_)_(*P*) can be deduced. A glossary, explaining the main mathematical symbols, is presented in Table 1.

**Table 1:** Glossary of main mathematical symbols.

**Parameters**	
*(m,n)*	Welding technique, welded material
*P*	Welding power, product of welding current and voltage
**Spectral and related quantities**	
*E*_sun_(λ)	Spectral irradiance of the Albuquerque summer sun, EN 13758‐1 9
*E*_(_ *_m_*_,_ *_n_*_)_(λ,*P*)	Welding arc’s spectral irradiance
*E*_eff(_ *_m_*_,_ *_n_*_)_(*P*)	*S*_uvh_(λ) weighted welding arc irradiance
*ε*_(_ *_m_*_,_ *_n_*_)_(λ)	*P* independent, normalized standard welding emission spectra
*τ_k_*(λ), ${\bar{\tau }}_{k}\pm s_{k}$	Transmittance, mean UVR transmittance and standard deviation
*S*_er_(λ)	CIE erythema reference action spectrum 7
*S*_uvh_(λ)	ICNIRP relative spectral effectiveness of UVR 13
**Ultraviolet protection factors**	
sUPF*_k_*	(solar) Ultraviolet Protection Factor according to Gies et al. 6
wUPF*_k_*_(_ *_m_*_,_ *_n_*_)_(*P*)	First stage wUPF
wUPF*_k_*_(_ *_m_*_,_ *_n_*_)_	Second stage, *P* independent wUPF based on *ε*_(_ *_m_*_,_ *_n_*_)_(λ)
${\bar{\text{wUPF}}}_{k}$	Final, (*m*,*n*) averaged wUPF
${\bar{\text{wUPF}}}_{k,250}$	Final wUPF, limited summation range λ ≥ 250 nm
rUPF*_k_*_,uvh_	Reduced wUPF, solely depending on *S*_uvh_(λ) and *τ_k_*(λ)
rUPF*_k_*_,uvh250_	Reduced wUPF, limited summation range λ ≥ 250 nm
rUPF*_k_*_,er_	Reduced, erythemally weighted UPF
rUPF*_k_*_,er290_	rUPF*_k_*_,er_, limited summation range λ ≥ 290 nm, equal to reduced solar UPF without *E*_sun_(λ)

## Materials and methods

### Fabrics

A selection of 17 white, gray, blue or yellow/beige fabrics, manufactured by kettelhack, was made varying partly with regard to composition (cotton co, polyester pes, and elastolefin eol), weave (plain 1|1, twill 2|1 and 3|1) and grammage *g* ranging from 145 to 350 gm^−2^. All clothing samples were labeled with an unsorted index *k*. No previous preparations were made, but the fabrics’ transmittances, τ*_k_*(λ), were determined in their purchasing condition. Table 2 summarizes the main parameters of the investigated textiles.

**Table 2:** Compilation of several textile characteristics for the *k* fabric samples used within this work.

	*k*	Composition	Weave	*g* (gm^‐2^)	Color	${\bar{\tau }}_{k}\pm s_{k}$ (%)
Figure 3 (a)	1	100co	plain 1|1	175	royal blue	2.2 ± 0.4
	2		twill 2|1	185	white	12 ± 3
	3		plain 1|1	190	white	18 ± 5
	4		twill 3|1	205	white	10 ± 2
	5		twill 2|1	210	white	10 ± 3
	6		twill 3|1	350	royal blue	(3 ± 3) 10^‐3^
Figure 3 (b)	8	50co50pes	plain 1|1	145	yellow/beige	9 ± 3
	9		twill 2|1	150	white	11 ± 3
	10		plain 1|1	175	light blue	7 ± 3
	11		twill 2|1	195	white	8 ± 2
	12		twill 2|1	215	white	9 ± 2
Figure 3 (c)	7	65co35pes	twill 2|1	245	light gray	1.3 ± 0.6
	13	35co65pes	twill 2|1	195	white	9 ± 2
	14		twill 3|1	350	dark blue	(3 ± 3) 10^‐3^
Figure 3 (d)	15	97co3eol	twill 2|1	210	white	9 ± 3
	16		twill 3|1	270	white	8 ± 2
	17	63co34pes3eol	twill 2|1	230	white	9 ± 2

Aside composition, weave, grammage *g*, and color, mean UVR transmittances are listed together with their standard deviations, ${\bar{\tau }}_{k}\pm s_{k}$.

These 17 fabrics are intended as basic material for workwear, worn by different groups of employees. Fabrics with low grammages, for example, *k* = 1,8 or 13, can be used to manufacture general workwear like the supervisor’s shirt, whereas the heavyweight cotton, *k* = 6, or blended fabrics, *k* = 14, are UV opaque and suitable for protective clothes like welders’ clothing. This fabric choice is appropriate for the calculation of a workplace specific welding UPF because the welding workplace as well as working areas nearby or passersby are considered.

Although some textile parameters vary, the selected samples do not represent a cross section of available materials for workwear. However, for the purpose of this work that is the demonstration of a new UPF concept for artificial UVR from welding arcs, the actual choice of fabrics plays a minor role. It would also have been possible to perform the wUPF calculation based on theoretical “test transmittances” with linear, exponential, cubic, etc. spectral distributions.

### Spectrophotometer

**Figure 3:**
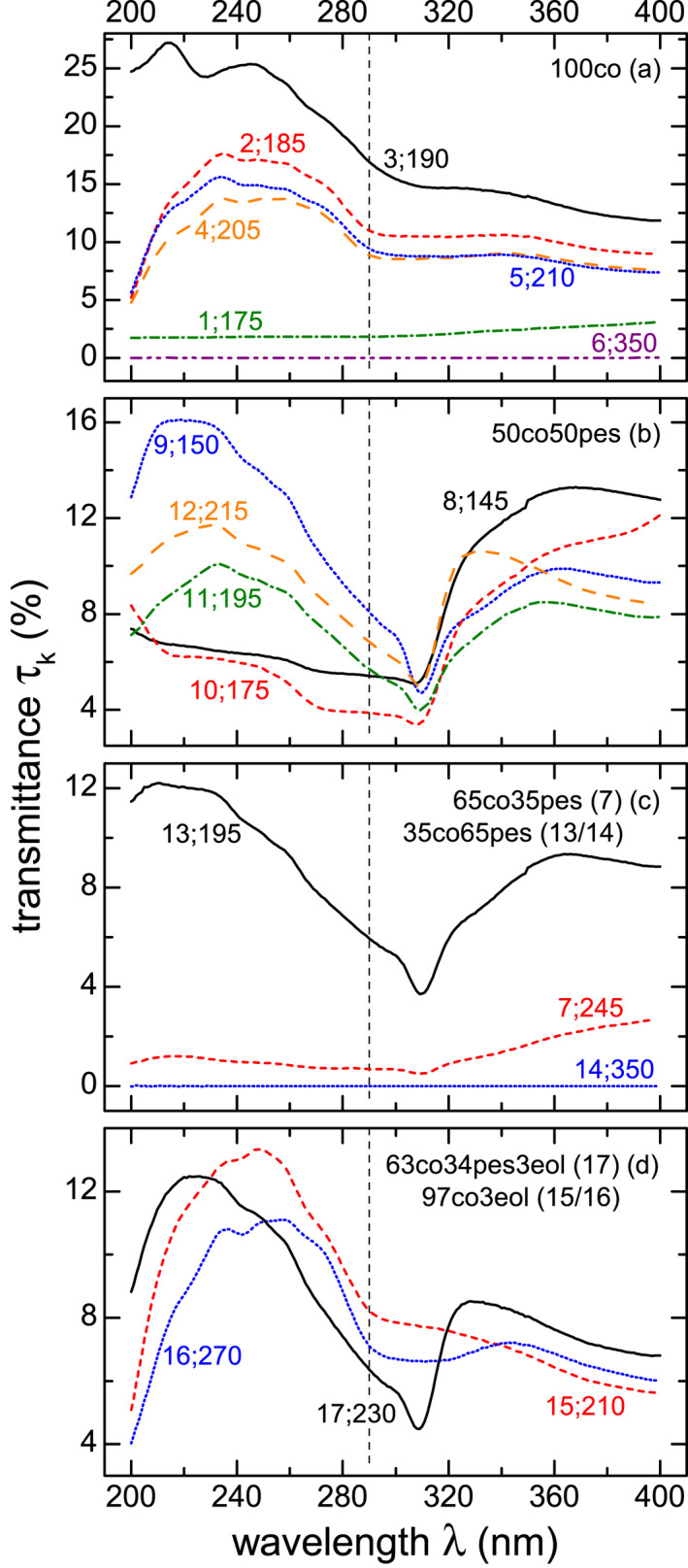
Workwear transmittances, *τ_k_*, in % of samples *k* = 1 to 17, see Table 2, separated by composition: (a) 100% cotton (co), (b) 50co and 50% polyester (pes), (c) 65co35pes and 35co65pes, as well as (d) 63co34pes and 97co, each with 3% elastolefin (eol). The fabric index *k* and the grammage *g* are given as paired numbers, that is, *k*;*g.*

A CARY 5000 UV‐Vis‐NIR spectrophotometer, in conjunction with a Diffuse Reflectance Accessory DRA‐2500, both from Agilent, was used to determine the spectral transmittances of the *k* fabrics, *τ_k_*(λ).The spectrophotometer was operated in double beam mode to account for UVR fluctuations caused by the deuterium source and the optical components. The reference beam was directed toward an SRS‐99 Spectralon diffuse reflectance standard, traceable to the National Institute of Standards and Technology (NIST), that was placed on the reference port of the DRA’s integrating sphere fully coated with Spectralon. The fabrics were mounted at the entrance port of the integrating sphere, so that all of the diffusely scattered radiation was detected. All transmittances, automatically zero baseline corrected by the spectrophotometer’s software, were measured from 2500 to 200 nm in steps of 1 nm with a scan rate of 600 nm min^−1^ (only the UV spectral region is depicted in Fig. 3). UV reflectances, *R*(λ), of selected fabrics were determined (not shown), but reflected UVR into the integrating sphere can be neglected for *τ_k_*(λ) measurements due to *R*(λ < 350 nm) < 5%. Subsequently, the *τ_k_*(λ) spectra were analyzed by calculating their mean UV transmittances, ${\bar{\tau }}_{k}$, and their standard deviations, *s_k_*. It must be noted that, although a preconditioning procedure was applied to the interior of the integrating sphere by the manufacturer, fluorescence still can influence transmittance spectra.

### Welding spectra

Spectral irradiances, *E*_(_ *_m_*_,_ *_n_*_)_(λ,*P*), were detected simultaneously at a distance of 50 cm by two spectroradiometers: a CAS140CT‐152 (Instrument Systems) and a BTS2048‐UV‐S (Gigahertz‐Optik). Due to fluctuations of the welding arc’s UVR emission, the measured welding spectra can differ significantly 21 leading to large uncertainties in the welding power dependent wUPF. Note that this unstable UVR emission is the dominant source of error compared with, for example, the spectroradiometer’s false light. Practically relevant combinations of welding power *P*, welding technique *m* (pulsed or standard metal active gas, (p−) MAG, Cold Metal Transfer, (p−) CMT, pulsed metal inert gas, p‐MIG, tungsten inert gas, TIG, plasma transferred arc, PTA) and welded material *n* (structural, S235, and stainless steel, CrNi, aluminum, Al) were investigated, only. A part of these welding spectra and additional measurement conditions are published in 12. The *S*_uvh_(λ) weighted actinic irradiances, *E*_eff(_ *_m_*_,_ *_n_*_)_(*P*), were calculated for each of the spectroradiometers’ *E*_(_ *_m_*_,_ *_n_*_)_(λ,*P*).

## RESULTS

### Fabric transmittances

Fabric transmittances, *τ_k_*(λ), presented in Fig. 3, are sorted by composition, that is, (a) 100% cotton (co), (b) 50:50 mixture of cotton and polyester (pes), (c) 65% or 35 % cotton with 35% or 65% polyester, respectively, and (d) cotton–polyester mixture with 3% elastolefin (eol).

One approach for a quantified comparison of *τ_k_*(λ) characteristics is given by the calculation of UV spectral region specific mean transmittances, ${\bar{\tau }}_{k}$, listed in Table 2. Many of the investigated fabric samples have high mean UVR transmittances. Both fabric samples with the highest grammage, *g* = 350 gm^−2^, *k* = 6 and 14, have the lowest ${\bar{\tau }}_{6,14}\;=\;\left(3\;\pm \;3\right)$ 10^−3^ % and can be considered as UV opaque. With decreasing *g*, an increase in ${\bar{\tau }}_{k}$ is observable, for example, for *k* = 2 and 5 (100co, twill 2|1, white) or *k* = 9 and 11 (50co50pes, twill 2|1, white), but the effects are small and are within the uncertainty ranges. In contrast, fabric *k* = 1, with a grammage of 175 gm^‐2^, only has a ${\bar{\tau }}_{1}$ = (2.2 ± 0.4) %. In general, dark (blue) colored fabrics, *k* = 1, 6 and 14, have smaller ${\bar{\tau }}_{k}$ values than white ones, in accordance with 11. Because of the limited sample number, it is difficult to draw further conclusions regarding, for example, weave.

### Welding power dependence

An increase in welding power is accompanied by more intense emission lines in the UV spectrum of the welding arc. The spectral distribution, however, usually remains unaltered (see section standard emission spectra) as the additional energy, due to the *P* increase, is insufficient to excite further emission lines. Consequently, the wUPF calculation in Eq. (2) should be welding power independent. Verifying this hypothesis, wUPF*_k_*_(_ *_m_*_,_ *_n_*_)_(*P*) are calculated with welding arc emission spectra, *E*_(_ *_m_*_,_ *_n_*_)_(*λ*,*P*), in conjunction with *S*_uvh_(λ) weighted UV irradiances, *E*_eff(_ *_m_*_,_ *_n_*_)_(*P*), for several welding techniques *m* and three different materials *n*.

The wUPF determination according to Eq. (2) was performed for four fabric samples with selected (*m*,*n*) combinations, only. At least 8 different welding powers, that is, 16 spectra (two spectroradiometers were used), were examined for each of the 14 (*m*,*n*) combinations from 12, so a total number of 16 × 14 = 224 *E*_(_ *_m_*_,_ *_n_*_)_(λ,*P*) would have to be analyzed. In conjunction with the 17 fabric samples, the calculation effort would increase to 3808 wUPFs. Reducing this number, pulsed MAG welding of construction steel (p‐MAG, S235) was chosen because of its linear actinic irradiance power dependence, its large number of accessible welding powers and the highest *E*_eff(_ *_m_*_,_ *_n_*_)_(*P*) values recorded in 12. In contrast, the power dependent actinic irradiances for CMT welding of S235 (CMT, S235) can be well described by a sigmoidal function. The third and fourth examined (*m*,*n*) combinations are pulsed MIG welding of aluminum (p‐MIG, Al) and TIG welding of stainless steel (TIG, CrNi) that were selected because of the different spectral distributions of their arc spectra with regard to S235 (see Fig. 2). Fabric samples *k* = 1 and 3 have identical compositions and weaves, comparable grammages, but their mean transmittances differ with ${\bar{\tau }}_{1}$ = (2.2 + 0.4) % and ${\bar{\tau }}_{3}$ = (18 + 5) %, see Table 2. In relation to these two fabrics, sample *k* = 7 varies in composition, weave, grammage and color, but ${\bar{\tau }}_{7}$ = (1.3 ± 0.6) % is similar to ${\bar{\tau }}_{1}$. Fabric sample *k* = 8 was chosen because of its yellow/beige color and the medium ${\bar{\tau }}_{8}$ = (9 ± 3) %.

**Figure 4:**
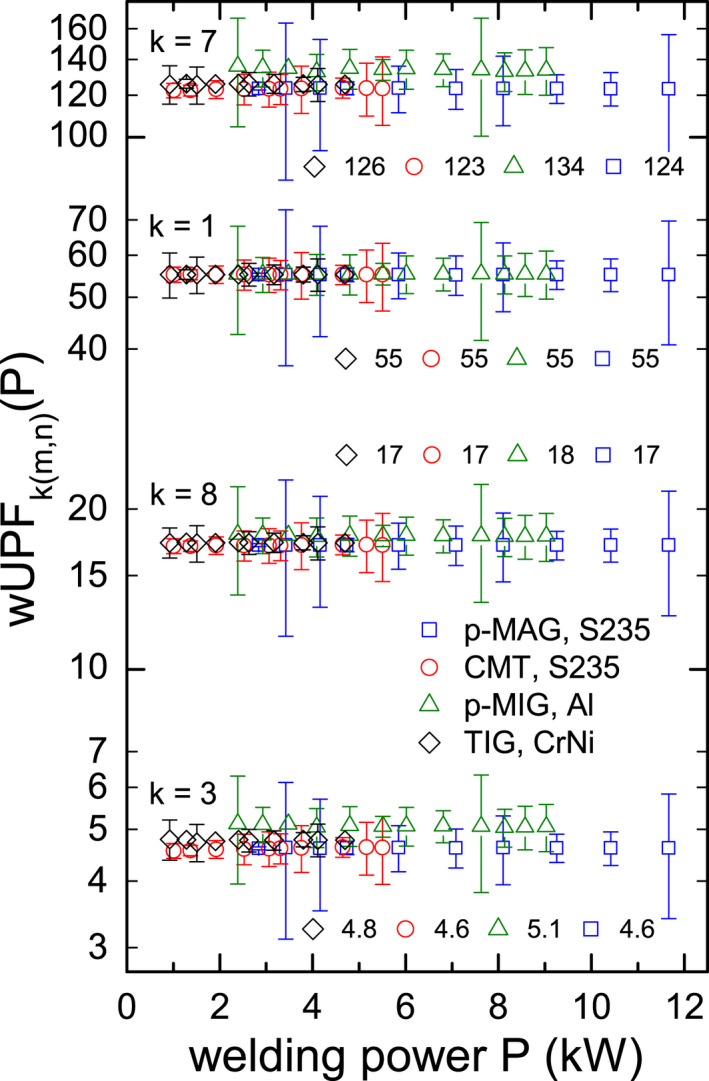
Semi‐logarithmic presentation of welding power dependent wUPF*_k_*_(_ *_m_*_,_ *_n_*_)_(*P*) calculated according to Eq. (2) for fabric samples *k* = 1, 3, 7 and 8 with pulsed MAG (*m* = p‐MAG, squares) and standard CMT welding (*m* = CMT, circles), both of structural steel (*n* = S235), with pulsed MIG welding of aluminum (*m* = p‐MIG, *n* = Al, triangles), and with TIG welding of stainless steel (*m* = TIG, *n* = CrNi, diamonds). Error bars result from two individual *E*_(m,n)_(λ,*P*) measurements 12. Power independent, averaged wUPF*_k_*_(_ *_m_*_,_ *_n_*_)_(*P*) values are given as numbers. Note that these averaged values are not equal to ${\bar{\text{wUPF}}}_{k}$.

Figure 4 depicts the corresponding ${\text{wUPF}}_{k\left(m,n\right)}\left(P\right)$ values. Error bars result from Gaussian error propagation of uncertainties in $E_{\left(m,n\right)}\left(\lambda ,P\right)$ originating from two independent welding spectra measurements, see section welding spectra. As suspected in the beginning of this section, no *P* dependence exists for the presented 16 ${\text{wUPF}}_{k\left(m,n\right)}\left(P\right)$. Their averaged values are given as numbers in Fig. 4 and show accordance for *k* = 1 and 8. Without considering their ${\text{wUPF}}_{k\left(m,n\right)}\left(P\right)$ error bars, fabric samples *k* = 3 and 7 have slightly increased averaged wUPF values. Linear (p‐MAG, S235) and sigmoidal (CMT, S235) mathematical $E_{\text{eff}\left(m,n\right)}\left(P\right)$ descriptions do not influence the wUPF. The effect of varying spectral distributions, that is (*m*,*n*) combinations, is weak, indicating that the use of normalized standard emission spectra might be appropriate to describe the wUPF.

### Standard welding emission spectra

By normalizing a total number of 139 power dependent welding spectra, each to its spectral irradiance maximum, and subsequently averaging certain (*m*,*n*) groups, standard welding emission spectra can be determined, see Fig. 2. There are no differences in the spectral distributions of (a) the normalized standard and p‐MAG welding spectra of stainless steel, (e) the normalized spectral irradiances of p‐CMT and p‐MIG welding of aluminum, denoted as p‐GMAW, Al, and those of (p−) MAG and (p−) CMT welding of structural steel, denoted as GMAW, S235, see Fig. 1 (b). Overall, 7 standard emission spectra, $\varepsilon_{\left(m,n\right)}\left(\lambda \right)$, result from the original 139 power dependent ones.

Briefly, the GMAW and PTA, S235, as well as the (p−) MAG, CrNi standard emission spectra look similar due to largely identical chemical compositions of the welded materials. In contrast, comparing Fig. 2 (a) with (b) or (c) with (d) reveals significant spectral differences depending on welding technique *m*. However, it is not the purpose of this work to give a detailed analysis of several welding spectra that can be found, for example, in 12. Based upon these seven standard emission spectra, Eq. (2) can be re‐written according to

(3) $${\text{wUPF}}_{k\left(m,n\right)}=\frac{\sum_{200\;\text{nm}}^{400\;\text{nm}}\;\varepsilon_{\left(m,n\right)}\left(\lambda \right)\;S_{\text{uvh}}\left(\lambda \right)\;\Delta \lambda }{\sum_{200\;\text{nm}}^{400\;\text{nm}}\;\varepsilon_{\left(m,n\right)}\left(\lambda \right)\;S_{\text{uvh}}\left(\lambda \right)\;\tau_{k}\left(\lambda \right)\;\Delta \lambda }$$

Note that Eq. (3) is no longer a function of welding power due to the replacement of $E_{\left(m,n\right)}\left(\lambda ,P\right)$ by $\varepsilon_{\left(m,n\right)}\left(\lambda \right)$, that is, grouping, normalizing and averaging several welding power dependent spectra. At a first glance, Eq. (3) does not seem to be a step forward; however, the number of wUPFs that must be calculated reduces from 3808 to 7 × 17 = 119.

Although some of the determined standard emission spectra vary significantly with regard to their spectral distributions, there is no effect of welding technique *m* and welded material *n* on the wUPF; thus, the ${\text{wUPF}}_{k\left(m,n\right)}$ can be averaged. These mean values, denoted as ${\bar{\text{wUPF}}}_{k}$, represent the final welding UPFs and are listed in Table 3 for all *k* fabrics. The standard deviations are *s* ≤ 5 % except for both fabrics with the highest grammages and the lowest UVR transmittances, that is, *k* = 6 and 14. A comparison of ${\bar{\text{wUPF}}}_{k}$ with the corresponding sUPF*_k_* will be presented in the discussion.

**Table 3:** List of final welding UPFs with standard deviations, ${\bar{\text{wUPF}}}_{k}\pm s$, reduced welding UPFs, rUPF*_k_*_,uvh_, their alternatives spectrally limited to λ ≥ 250 nm, ${\bar{\text{wUPF}}}_{k,250}\pm s$ and rUPF*_k_*_,uvh250_, respectively, solar UPFs, sUPF*_k_*, reduced solar UPFs, rUPF*_k_*_,er290_, and reduced erythemally weighted UPFs, rUPF*_k_*_,er_, of the *k* fabrics.

*k*	${\bar{\text{wUPF}}}_{k}\pm s$	rUPF*_k_*_,uvh_	${\bar{\text{wUPF}}}_{k,250}\pm s$	rUPF*_k_*_,uvh250_	sUPF*_k_*	rUPF*_k_*_,er290_	rUPF*_k_*_,er_
1	55.22 ± 0.07 (0.1)	55	55.15 ± 0.06 (0.1)	55	50	54	55
2	7.0 ± 0.3 (4)	6.9	7.1 ± 0.3 (4)	7.1	9.6	9.4	7.2
3	4.8 ± 0.2 (3)	4.7	4.9 ± 0.1 (3)	5.0	6.7	6.3	5.0
4	8.5 ± 0.3 (4)	8.6	8.7 ± 0.3 (4)	8.7	12	12	8.9
5	8.0 ± 0.3 (4)	8.0	8.2 ± 0.3 (4)	8.3	11	11	8.4
6	(44 ± 5)×10^3^ (13)	42 × 10^3^	(57 ± 5)×10^3^ (9)	61 × 10^3^	40 × 10^3^	41 × 10^3^	41 × 10^3^
7	127 ± 3 (2)	124	133 ± 3 (2)	134	135	150	133
8	17.4 ± 0.2 (1)	17	17.7 ± 0.2 (1)	18	15	19	18
9	9.3 ± 0.5 (5)	9.0	9.8 ± 0.5 (5)	9.9	16	14	10
10	22.3 ± 0.8 (4)	22	23.4 ± 0.8 (3)	23	21	26	23
11	13.4 ± 0.6 (4)	13	13.9 ± 0.6 (4)	14	20	19	14
12	11.5 ± 0.4 (4)	11	11.9 ± 0.4 (4)	12	15	16	12
13	12.6 ± 0.6 (5)	12	13.3 ± 0.6 (5)	13	20	19	14
14	(63 ± 3)×10^3^ (6)	60 × 10^3^	(64 ± 3)×10^3^ (5)	60 × 10^3^	59 × 10^3^	56 × 10^3^	61 × 10^3^
15	9.4 ± 0.4 (4)	9.3	9.6 ± 0.4 (4)	9.7	13	13	9.8
16	10.6 ± 0.4 (4)	11	10.7 ± 0.4 (4)	11	15	15	11
17	11.7 ± 0.6 (5)	11	12.3 ± 0.6 (5)	12	18	17	12

The percentage standard deviations of ${\bar{\text{wUPF}}}_{k}$ and ${\bar{\text{wUPF}}}_{k,250}$ are given in parenthesis.

### Reduced wUPF

As demonstrated in the previous section, the calculation of welding UPFs does not depend on the wavelength distribution of normalized welding spectra, $\varepsilon_{\left(m,n\right)}\left(\lambda \right)$, for different (*m*,*n*) combinations. Consequently, the wUPFs can be averaged resulting in ${\bar{\text{wUPF}}}_{k}$, see Table 3. This finding indicates that *ε*_(_ *_m_*_,_ *_n_*_)_(λ) might be regarded as a constant factor in Eq. (3), thus reducing the wUPF*_k_*_(_ *_m_*_,_ *_n_*_)_ formula by removing the arc’s spectral irradiance:

(4) $${\text{rUPF}}_{k,\text{uvh}}=\frac{\sum_{200\;\text{nm}}^{400\;\text{nm}}S_{\text{uvh}}\left(\lambda \right)\;\Delta \lambda }{\sum_{200\;\text{nm}}^{400\;\text{nm}}S_{\text{uvh}}\left(\lambda \right)\;\tau_{k}\left(\lambda \right)\;\Delta \lambda }=45.8\;\text{nm}\;\times \;\tau_{k,\text{uvh}}^{-1}$$

The use of ICNIRP’s relative spectral effectiveness for UVR hazards 13 is noted as “uvh” subscript. The summation in the numerator results in a constant value of 45.8 nm. The denominator can be seen as a biologically effective transmittance *τ* *_k_*_,uvh_. With Eq. (4), the calculation of formerly 3808 wUPFs has decreased to a total of #*k* = 17 reduced welding UPFs (rUPF), listed in Table 3. All of them are sufficiently accurate to represent ${\bar{\text{wUPF}}}_{k}\pm s$.

## DISCUSSION

### Welding UPF comparison

#### Reduced to welding UPF

**Figure 5:**
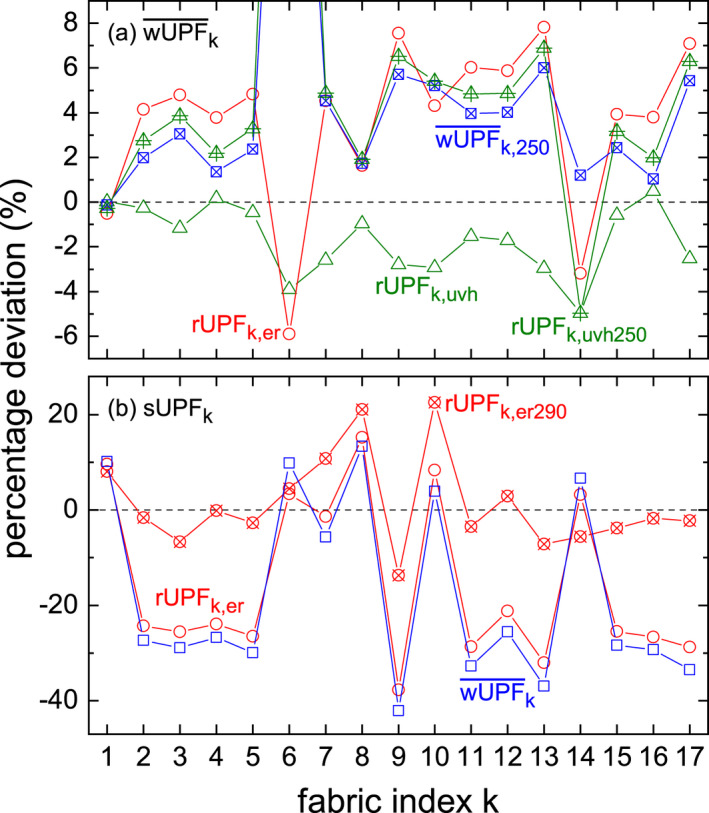
(a) Percentage deviations of reduced welding UPFs (triangles), rUPF*_k_*_,uvh_, Eq. (4), reduced erythemally weighted UPFs (circles), rUPF*_k_*_,er_, spectrally limited alternatives of the welding UPF and its associated reduced version, ${\bar{\text{wUPF}}}_{k,250}$ (crossed squares) and rUPF*_k_*_,uvh250_ (crossed triangles), respectively, with regard to the averaged, welding power and (*m*,*n*) independent wUPFs, ${\bar{\text{wUPF}}}_{k}$. (b) Percentage deviations of ${\bar{\text{wUPF}}}_{k}$ (squares), reduced solar UPFs (crossed circles), rUPF*_k_*_,er290_, Eq. (5), and rUPF*_k_*_,er_ (circles) with regard to the solar UPF, sUPF*_k_*, according to Eq. (1). The dashed lines visualize zero percentage deviation.

Percentage deviations of the *k* reduced rUPF*_k_*_,uvh_ with regard to their ${\bar{\text{wUPF}}}_{k}$ are depicted in Fig. 5 (a). All values are in the range of −5% to 0.5%. Only three of the 17 fabrics (*k* = 1, 4, and 16) have slightly higher rUPF*_k_*_,uvh_ than compared to their welding UPFs, whereas all other textiles have negative percentage deviations. It must be remarked that neither the propagation of the ${\bar{\text{wUPF}}}_{k}$ uncertainty nor the $\tau_{k}\left(\lambda \right)$ measurement error have been considered in Fig. 5 (a); thus, no valid conclusions can be drawn regarding the signs of those fabrics that are close to zero percentage deviation. Considering ± *s* the reduced wUPFs agree with ${\bar{\text{wUPF}}}_{k}$, see Table 3. It can be concluded that, in case of artificial UVR from welding arcs, the calculation of wUPFs for workwear, and thereby the protective effect of these textiles against such UVR, mainly depends on the *S*_uvh_(λ) action spectrum and the fabric’s transmittance.

#### Welding to solar UPF

Table 3 also shows the sUPFs for the *k* fabrics, calculated according to Eq. (1). The percentage deviations of ${\bar{\text{wUPF}}}_{k}$ with regard to sUPF*_k_*, shown in Fig. 5 (b), are within −44% to 12%. These values are high, but one has to keep in mind that the prerequisites for the calculation of both UPFs differ with regard to the applied action spectra, accompanied by changed summation ranges, and the UVR sources (solar versus artificial UVR). A closer look at Fig. 5 (b), in combination with Table 3, reveals that the ${\bar{\text{wUPF}}}_{k}$ is about 4% to 13% higher than the corresponding sUPF*_k_* for most of the colored fabric samples, *k* = 1, 6, 8, 10 and 14, whereas all white textiles have −26% to −44% smaller ${\bar{\text{wUPF}}}_{k}$. The light gray fabric *k* = 7, with its “color” being spectrally close to white, is the only exception. It is unclear, why color, originating from visible wavelengths, has such an effect on the welding or solar UPF. Further research will have to be conducted based on a larger fabric sample number.

### Reduced solar and erythemally weighted UPF

The accordance of reduced with their welding UPFs, that is, rUPF*_k_*_,uvh_ being close to ${\bar{\text{wUPF}}}_{k}$, leads to the question, whether a reduced solar UPF will also agree with the original calculation according to Eq. (1). Following Eq. (4),

(5) $${\text{rUPF}}_{k,\text{er}290}=\frac{\sum_{290\;\text{nm}}^{400\;\text{nm}}S_{\text{er}}\left(\lambda \right)\;\Delta \lambda }{\sum_{290\;\text{nm}}^{400\;\text{nm}}S_{\text{er}}\left(\lambda \right)\;\tau_{k}\left(\lambda \right)\;\Delta \lambda }=12.7\;\text{nm}\;\times \;\tau_{k,\text{er}290}^{-1}$$

no longer considers the solar spectral irradiance, *E*_sun_(λ). The “290” subscript for rUPF*_k_*_,er290_ indicates that the summation limits stay unchanged, although *S*_er_(λ) also reflects relative spectral erythema effectiveness for 250 nm ≤ λ < 290 nm. The sum in the numerator is constant with 12.7 nm. Again, *τ*_*k*,er290_ can be thought of as biologically effective UV transmittance, now depending on the *S*_er_(λ) weighting function. Table 3 lists all of the rUPF*_k_*_,er290_ values.

#### Reduced solar to sUPF

Figure 5 (b) shows the *k* dependent percentage deviations of rUPF*_k_*_,er290_ with regard to sUPF*_k_*. These deviations range in between −7% and 8%, except for the colored fabric samples *k* = 7, 8, 10, and the white one, *k* = 9, see Table 2. Five of the six colored textiles, *k* = 1, 6, 7, 8 and 10, have positive percentage deviations, whereas that of *k* = 14 (dark blue, highest grammage) is negative with −6% (disregarding uncertainty analysis). Sample *k* = 12 is the only white fabric with a positive deviation, although it is comparably small with 3%. Again, as in the case of the ${\text{rUPF}}_{k,\text{uvh}}\text{-}{\bar{\text{wUPF}}}_{k}$‐comparison, color seems to play a role for rUPF*_k_*_,er290_ deviations regarding sUPF*_k_*.

#### Reduced erythemal to solar and welding UPF

Enlarging the summation in Eq. (5) to the full wavelength range of *S*_er_(λ), that is, 250 ‐ 400 nm, leads to an increase of the constant numerator value from 12.7 to 52.7 nm. The corresponding reduced erythemally weighted UPF, rUPF*_k_*_,er_ (now without “290” subscript), listed in Table 3, considers all wavelengths that are able to cause erythema in human skin, according to CIE. Their percentage deviations regarding sUPF*_k_* are presented in Fig. 5 (b) and follow closely the ${\bar{\text{wUPF}}}_{k}$ deviations; hence, an accurate description of solar UPFs with rUPF*_k_*_,er_ is not possible. However, percentage deviations of rUPF*_k_*_,er_ regarding ${\bar{\text{wUPF}}}_{k}$, shown in Fig. 5 (a), are smaller than + 8%, except for *k* = 1 and for both high grammage fabrics, *k* = 6 and 14, that show negative deviations. Although UVC radiation below 250 nm has not been taken into account for the rUPF*_k_*_,er_ calculation, reduced erythemally weighted UPFs approach ${\bar{\text{wUPF}}}_{k}$, when considering the additional 40 nm, that is wavelengths 250 nm ≤ λ < 290 nm.

### Spectrally limited welding UPFs

UVC radiation is absorbed partly by the human skin so that, at least to some extent, an epidermal self‐protection exists. Additionally, UVC measurements can be flawed because of the appearance of stray light (or better false light), although modern spectroradiometers are usually equipped with appropriate filters or use stray light matrices, and the welding arc itself is by far the dominant source of measurement uncertainty 21. It is a major concern to include UVC wavelengths below 250 nm in the proposed wUPF, but a spectrally limited version, that is, restricting the summation range in Eq. (3) to 250 nm ≤ λ ≤ 400 nm, referring to the CIE action spectrum, and subsequent (*m*,*n*) averaging leading to ${\bar{\text{wUPF}}}_{k,250}$, can be contemplated.

In Fig. 5 (a), percentage deviation of ${\bar{\text{wUPF}}}_{k,250}$ and its associated reduced version according to Eq. (4), rUPF*_k_*_,uvh250_, are presented with regard to the “full” wUPF. Similar to the findings for rUPF*_k_*_,er_, most of the percentage deviations are positive and smaller than + 7% except for the high grammage fabrics *k* = 6 and 14. Textile *k* = 1 is close to zero, but no valid conclusion can be drawn without a thorough uncertainty analysis. Disregarding fabric sample *k* = 6, percentage deviations of ${\bar{\text{wUPF}}}_{k,250}$ and rUPF*_k_*_,uvh250_ follow those of rUPF*_k_*_,er_. Comparing both spectrally limited welding UPFs with the reduced erythemally weighted UPF, Table 3, reveals agreement among each other, in accordance what CIE found for sUPFs derived with either *S*_er_(λ) or *S*_uvh_(λ) 19. Overall, omitting wavelengths λ < 250 nm is accompanied by slightly higher UPFs with regard to ${\bar{\text{wUPF}}}_{k}$.

### Occupational safety and health

Welders are usually well protected against the arc’s UVR by their special clothing. It has to fulfill certain requirements regarding flying sparks and thermal radiation 22; thus, it is often manufactured with high grammage fabrics (or leather) typically having low UVR transmittances. But people working nearby or passing welding workplaces, for example, forklift drivers, will avoid wearing these heavy and often uncomfortable protective clothing. Instead, they will dress with general workwear, for example, with some of those listed in Table 2.

Assuming that workwear made of fabric *k* = 12, that would be labeled with an sUPF of 15, will be worn, an underestimation of the potentially detrimental effects of artificial UVR might appear. For example, the actinic irradiance for p‐MAG welding of S235 with *P* = 9 kW at a distance of 4 m to the welding arc is given by *E*_eff_ = 10 Wm^−2^ 12. The erythemally weighted irradiance is *E*_er_ = 12 Wm^−2^. In conjunction with the ICNIRP ELV, *H*_eff_ = 30 Jm^−2^ 13, and the minimum radiant exposure for perceptible skin reddening, 1 MED = 200 Jm^‐2^, maximum permissible exposure durations, *t*_eff_ = 3 s and *t*_er_ ≈ 17 s, can be calculated. Then, for fabric sample *k* = 12, sUPF_12_ = 15 yields a maximum textile photoprotection time of 4 min 10 s, whereas the exposure duration based on ${\bar{\text{wUPF}}}_{12}=11$ is only 33 s. Never intended to assess photoprotection against artificial UVR, sUPFs are not appropriate to be applied for workwear used at welding workplaces.

## Conclusion

Based on the idea of the solar ultraviolet protection factor (sUPF), a modified welding UPF (wUPF) was proposed for workwear exposed to artificial UVR from welding arcs. Replacing the sun’s UV spectral irradiance and the CIE erythema weighting function by power dependent welding arc spectra and the ICNIRP relative spectral effectiveness of UVR, respectively, allowed an appropriate adaption of the sUPF equation. The resulting wUPF is intended for textiles worn by passersby or employees at adjacent workplaces, because the welder himself is usually well protected by its special workwear.

The welding power dependence of spectral UV irradiances, emitted from diverse welding arcs, has been considered, but the wUPF was found to be welding power independent. Consequently, normalized standard welding emission spectra were derived demonstrating that welding technique and welded material only have a minor effect on the wUPF; hence, averaged values can be used. This finding led to a reduced welding UPF (rUPF), solely taking into account the textile’s UV transmittance and the ICNIRP action spectrum, and being sufficiently accurate to describe the wUPF.

For the nonrepresentative choice of 17 workwear fabrics, wUPFs were found to deviate by about −44% to 12% from their sUPFs. The reduced solar UPF, rUPF*_k_*_,er290_, was not appropriate to serve as an approximation for all sUPFs. An “enlarged” version, rUPF*_k_*_,er_, considering all erythemally relevant UV wavelengths, 250 to 400 nm, according to CIE, failed to describe the solar UPF, but deviated by less than 8% from the wUPF. Omitting wavelengths λ < 250 nm was accompanied by higher (reduced) UPF values with regard to the wUPF. A comparison of maximum permissible exposure durations, based on either the ICNIRP ELV or the 1 MED erythema ELV surrogate, demonstrated the necessity for a careful handling of sUPFs at workplaces where employees are exposed to artificial UVR from welding arcs.

Finally, it is worth to emphasize the huge impact of the sUPF and its great success. The considerations of this work do not aim at a revision of the sUPF formula, but are intended to provide a trigger for the application of different kinds of UPFs at workplaces with artificial UVR. Therefore, further research will have to be carried out regarding other artificial UVR sources with varying spectral irradiances, and a larger fabric sample number to unravel, for example, the effect of color on wUPFs. Then, the concept of reduced UPFs will also be able to be evaluated thoroughly, maybe with additional weighting functions.
